# Opening the “black box” of *nodD3*, *nodD4* and *nodD5* genes of *Rhizobium tropici* strain CIAT 899

**DOI:** 10.1186/s12864-015-2033-z

**Published:** 2015-10-26

**Authors:** Pablo del Cerro, Amanda Alves Paiva Rolla-Santos, Douglas Fabiano Gomes, Bettina Berquó Marks, María del Rosario Espuny, Miguel Ángel Rodríguez-Carvajal, María Eugenia Soria-Díaz, André Shigueyoshi Nakatani, Mariangela Hungria, Francisco Javier Ollero, Manuel Megías

**Affiliations:** Departamento de Microbiología, Facultad de Biología, Universidad de Sevilla, Avda. Reina Mercedes, 6 Apdo Postal 41012, Sevilla, Spain; Embrapa Soja, C.P. 231, 86001-970 Londrina, Paraná Brazil; Departamento de Química Orgánica, Facultad de Química, Universidad de Sevilla, Apdo Postal 553, 41071 Sevilla, Spain; Centro de Investigación Tecnología e Innovación de la Universidad de Sevilla (CITIUS), Avda. Reinas Mercedes, 4B, 41012 Sevilla, Spain

**Keywords:** Biological nitrogen fixation, LCO, *nodD* gene, Nod factors, Symbiosis

## Abstract

**Background:**

Transcription of nodulation genes in rhizobial species is orchestrated by the regulatory *nodD* gene. *Rhizobium tropici* strain CIAT 899 is an intriguing species in possessing features such as broad host range, high tolerance of abiotic stresses and, especially, by carrying the highest known number of *nodD* genes—five—and the greatest diversity of Nod factors (lipochitooligosaccharides, LCOs). Here we shed light on the roles of the multiple *nodD* genes of CIAT 899 by reporting, for the first time, results obtained with *nodD3*, *nodD4* and *nodD5* mutants.

**Methods:**

The three *nodD* mutants were built by insertion of Ω interposon. Nod factors were purified and identified by LC-MS/MS analyses. In addition, *nodD1* and *nodC* relative gene expressions were measured by quantitative RT-PCR in the wt and derivative mutant strains. Phenotypic traits such as exopolysaccharide (EPS), lipopolysaccharide (LPS), swimming and swarming motilities, biofilm formation and indole acetid acid (IAA) production were also perfomed. All these experiments were carried out in presence of both inducers of CIAT 899, apigenin and salt. Finally, nodulation assays were evaluated in up to six different legumes, including common bean (*Phaseolus vulgaris* L.).

**Results:**

Phenotypic and symbiotic properties, Nod factors and gene expression of *nodD3, nodD4* and *nodD5* mutants were compared with those of the wild-type (WT) CIAT 899, both in the presence and in the absence of the *nod*-gene-inducing molecule apigenin and of saline stress. No differences between the mutants and the WT were observed in exopolysaccharide (EPS) and lipopolysaccharide (LPS) profiles, motility, indole acetic acid (IAA) synthesis or biofilm production, either in the presence, or in the absence of inducers. Nodulation studies demonstrated the most complex regulatory system described so far, requiring from one (*Leucaena leucocephala, Lotus burtii*) to four (*Lotus japonicus*) *nodD* genes. Up to 38 different structures of Nod factors were detected, being higher under salt stress, except for the *nodD5* mutant; in addition, a high number of structures was synthesized by the *nodD4* mutant in the absence of any inducer. Probable activator (*nodD3* and *nodD5*) or repressor roles (*nodD4*), possibly via *nodD1* and/or *nodD2*, were attributed to the three *nodD* genes. Expression of *nodC*, *nodD1* and each *nodD* studied by RT-qPCR confirmed that *nodD3* is an activator of *nodD1*, both in the presence of apigenin and salt stress. In contrast, *nodD4* might be an inducer with apigenin and a repressor under saline stress, whereas *nodD5* was an inducer under both conditions.

**Conclusions:**

We report for *R. tropici* CIAT 899 the most complex model of regulation of nodulation genes described so far. Five *nodD* genes performed different roles depending on the host plant and the inducing environment. Nodulation required from one to four *nodD* genes, depending on the host legume. *nodD3* and *nodD5* were identified as activators of the *nodD1* gene, whereas, for the first time, it was shown that a regulatory *nodD* gene—*nodD4*—might act as repressor or inducer, depending on the inducing environment, giving support to the hypothesis that *nodD* roles go beyond nodulation, in terms of responses to abiotic stresses.

**Electronic supplementary material:**

The online version of this article (doi:10.1186/s12864-015-2033-z) contains supplementary material, which is available to authorized users.

## Background

The association of rhizobial strains and legumes represents one of the most perfect symbiotic interactions, in which a sophisticated machinery has been developed in both partners for millions of years, now contributing the highest inputs of nitrogen on Earth [1–4]. A fascinating step in the symbiosis is represented by the molecular signal dialogue established between the compatible partners, starting with the message sent with the exudation of molecules—mainly flavonoids—from the host legume, and replied with the synthesis of lipochitooligosaccharides (LCOs)—also known as Nod factors—by the rhizobium [5–10]. The “maestro” that orchestrates this symphony in the bacterium is the regulatory *nodD* gene, constitutively expressed and responsible for initiating the transcriptions of the remaining nodulation genes [9–12].

*Rhizobium tropici* is abundantly found in tropical acid soils of South America; its main characteristics are high tolerance of environmental stresses and ability to nodulate a broad range of legumes, the most economically important being common bean (*Phaseolus vulgaris* L.) [13–16]. Probably the most intriguing feature of the common bean-*R. tropici* symbiosis is the abundance of flavonoid *nod*-gene inducers released by the host legume [17, 18], and the synthesis of the largest known variety of Nod factors by *R. tropici* CIAT 899 [19–23]. Also unique are the observations that *R. tropici* CIAT 899 is capable of producing LCOs under abiotic stresses—such as acidic and saline conditions—in the absence of plant-molecular signals [20–23], and interestingly, some LCOs are produced even in the absence of saline stress and flavonoids [23].

Rhizobial species described so far have one to five regulatory *nodD* genes. *R. tropici* CIAT 899 and closely related species carrying the symbiovar tropici (*R. leucaenae* CFN 299, *R. freirei* PRF 81) possess the highest numbers, with five copies of *nodD* genes [24, 25]. Elucidating the roles of the five *nodD* genes of CIAT 899 may help to understand their protagonism in host-range characteristics and in the strategies that the strain uses to circumvent abiotic stresses. It may also contribute to gaining a better understanding of the evolution of symbiotic interactions, since *R. tropici* has a strong resemblance to the pathogen *Agrobacterium* in terms of genes and proteins [25, 26].

As a first study, we shed light on the roles of *nodD1* and *nodD2* of *R. tropici* CIAT 899, with data related to the activation/repression of nodulation genes, their role in host range and showing that these two genes have functions beyond nodulation [23]. Here we achieve improved understanding of the mechanisms controlling regulation of the *nodD* genes of CIAT 899, with studies of *nodD3*, *nodD4* and *nodD5* mutants.

## Results and discussion

### Gene localization and phenotypes *in vitro* of the wild type and mutant strains

**Fig. 1:**
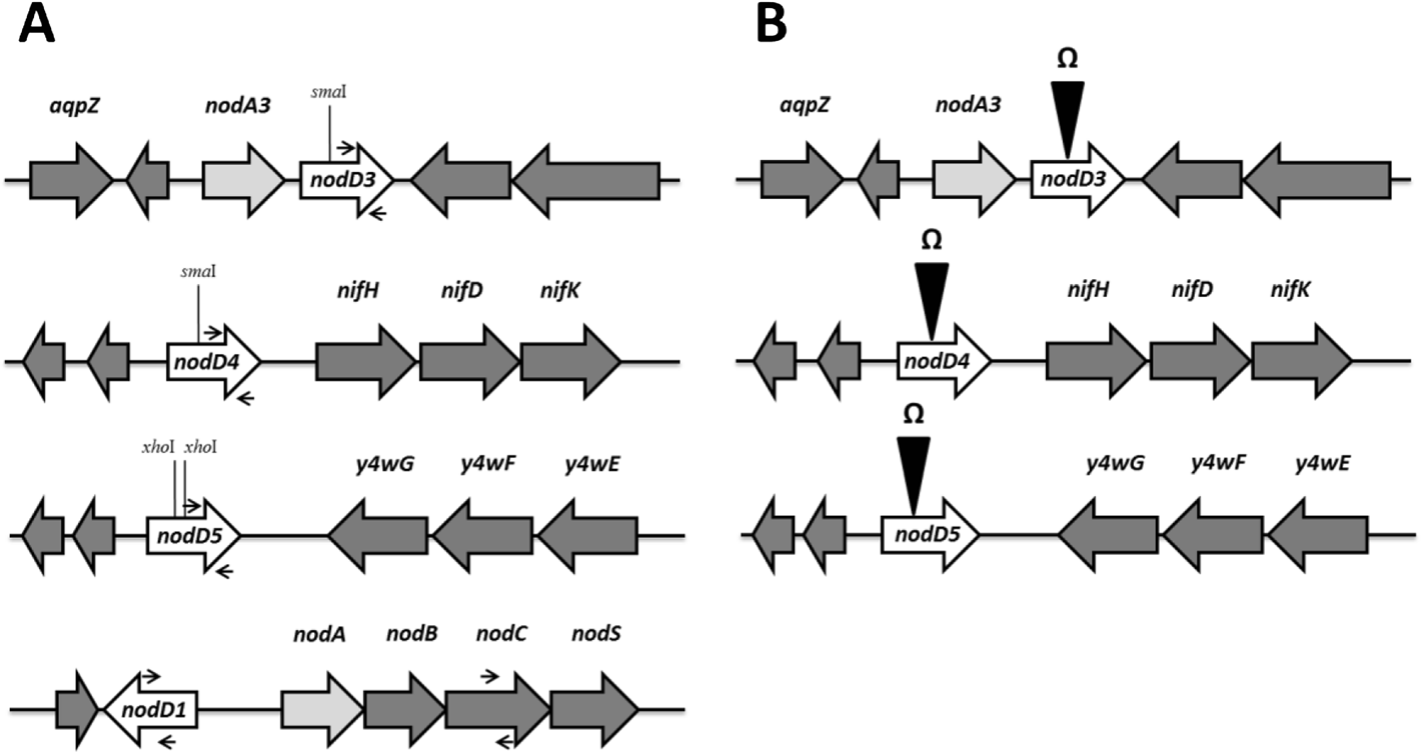
Gene neighborhood of *nodD3*, *nodD4* and *nodD5* genes and representation of the mutations. **a** Gene localizationin the symbiotic plasmid (pRtCIAT899b) of *R. tropici* strain CIAT 899 and location of primers used to perform RTqPCR experiments (dark arrows); **b** Schematic representation of the *nodD3*, *nodD4* and *nodD5* mutation

*nodD* gene nomenclature was used as defined for the genome of *R. tropici* strain CIAT 899 [25]. *nodA3* precedes *nodD3* and is close to an aquaporin; *nodD4* precedes the operon *nifHDK* and *nodD5* is downstream of an operon of hypothetical proteins (Fig. 1). *nodD3*, *nodD4* and *nodD5* genes correspond to the *CD5, CD21* and *CD29 nodD*-hybridizing regions of CIAT 899 described by van Rhijn et al. [24], respectively. Mutations in the *nodD3, nodD4* and *nodD5* genes were achieved as described in the Methods section. In general, evaluations were performed in the absence of any inducer (B^−^ medium) [20], in the presence of the *nod*-gene-inducing molecule apigenin (3.7 μM), or under salinity stress (NaCl 300 mM) that also induce the synthesis of Nod factors. The same treatments were used in our previous study with *nodD1* and *nodD2* mutants [23].

Some bacterial properties may be regulated via NodD proteins, and in our previous study we showed that both *nodD1* and *nodD2* have a constitutive suppression role on swarming motility and an activation effect on indole acetic acid (IAA) synthesis [23]. Here, we found no differences between the three mutants and the WT strain in exopolysaccharide (EPS) and lipopolysaccharide (LPS) profiles, swimming and swarming motilities, biofilm formation, or IAA synthesis (data not shown).

### Symbiotic phenotypes

Symbiotic phenotypes of the WT CIAT 899 and the mutant strains were verified in pots containing sterile substrate (Leonard jars). Previously, we reported that common bean requires both *nodD1* and *nodD2*, whereas *nodD1* was the main nodulation regulator of both leucaena [*Leucanea leucocephala* (Lam.) de Wit] and siratro [*Macroptilium atropurpureum* (DC.) Urb.] [23]. For comparison, these results are shown again in Table 1, together with the results obtained with *nodD3*, *nodD4* and *nodD5* mutants; in addition, the symbiotic properties of all five *nodD* mutants were assayed in two other host legumes, *Lotus burtii* Borsos and *Lotus japonicus* (Regel) K. Larsen.

**Table 1:** Plant responses (nodule number, n°/plant) and shoot dry weight (g/pl) to inoculation of common bean, leucaena, siratro, *Lotus japonicus* and *L. burtii* with *R. tropici* strain CIAT 899 and derivatives. Plants evaluated after 25 (common bean.), 42 days (leucaena and siratro), or 50 days (*Lotus* spp.) of growth under controlled conditions

Strains	*P. vulgaris*		*L. leucocephala*		*M. atropurpureum*		*L. japonicus*		*L. burtii*	
	Nodule number	Shoot dry weight	Nodule number	Shoot dry weight	Nodule number	Shoot dry weight	Nodule number	Shoot dry weight	Nodule number	Shoot dry weight
*R. tropici* CIAT 899	213 ± 52^a^	1.82 ± 0.64^a^	13 ± 4^a^	0.41 ± 0.03^a^	34 ± 8^a^	0.05 ± 0.01^a^	22 ± 9	0.064 ± 0.03	11 ± 5	0.04 ± 0.02
*nodD1* mutant	38 ± 11^*,a^	1.42 ± 0.35^a^	0 ± 0^*,a^	0.09 ± 0.01^*,a^	0 ± 0^*,a^	0.05 ± 0^a^	0 ± 0^*^	0.007 ± 0.004^*^	5 ± 2^*^	0.04 ± 0.02
*nodD2* mutant	95 ± 38^*,a^	1.03 ± 0.27^a^	10 ± 3^a^	0.36 ± 0.04^**,a^	24 ± 8^a^	0.05 ± 0^a^	30 ± 10	0.039 ± 0.013^*^	12 ± 5	0.03 ± 0.01
*nodD3* mutant	182 ± 25	1.80 ± 0.34	13 ± 3	0.39 ± 0.09	38 ± 12	0.05 ± 0	12 ± 4^*^	0.025 ± 0.014^*^	9 ± 4	0.04 ± 0.01
*nodD4* mutant	190 ± 24	1.84 ± 0.5	14 ± 3	0.37 ± 0.01^*^	29 ± 8	0.05 ± 0	17 ± 6	0.058 ± 0.052	12 ± 5	0.03 ± 0.02
*nodD5* mutant	179 ± 16	1.45 ± 0.68	12 ± 4	0.37 ± 0.01^*^	33 ± 7	0.05 ± 0.01	6 ± 2^*^	0.021 ± 0.011^*^	12 ± 2	0.05 ± 0.01
None	0 ± 0^*^	0.80 ± 0.25^*^	0 ± 0^*^	0.09 ± 0.01^*^	0 ± 0^*^	0.05 ± 0	0 ± 0^*^	0.008 ± 0.004^*^	0 ± 0^*^	0.01 ± 0^*^

^a^After [23]

^*^Data represent means ± SD (standard deviation) of 6 jars, each with two plants. *nodD3, nodD4* and *nodD5* mutant parameters were individually compared with the parental strain CIAT 899 parameters by using the Mann-Whitney non-parametric test. Values tagged by ^*^ and ^**^ are significantly different at the level α = 10 and 5 %, respectively

For the common bean, there were no statistical differences between the WT and the three mutants in terms of the nodulation or shoot dry weight (SDW) parameters, but we should mention that there was a decrease in nodulation, which might indicate effects of minor magnitude due to the mutations, with an emphasis on *nodD5*, where it also affected SDW. Leucaena and siratro also did not show differences in nodulation between the WT and the three mutants, but SDW of leucaena inoculated with *nodD4* and *nodD5* mutants was slightly, but significantly, lower (Table 1).

In relation to the nodulation of *Lotus* species, *nodD1* was the main regulator in *L. japonicus* and very important for *L. burtii*, whereas *nodD2* did not affect nodulation but resulted in a significative reduction in SDW of *L. japonicus*, indicating that it interferes with the efficiency of nitrogen fixation. In addition, for *L. japonicus*, a mutation in *nodD3* and *nodD5* also affected nodulation and SDW, whereas no effects were observed for *L. burtii* (Table 1).

From these data, we may conclude that full nodulation of common bean requires both *nodD1* and *nodD2* genes, but it is possible that *nodD3* and *nodD5* could make minor contributions that were not statistically detected in our experiments. Therefore, as in several other rhizobial species [27, 28] including the broadly nodulating *S. fredii* NGR 234 [29], *nodD1* is the main gene regulating nodulation of leucaena, siratro, *L. burtii* and *L. japonicus*. Siratro does not require any other *nodD* gene for nodulation, but a non-statistically significant decrease observed with the *nodD2* mutant should be more fully investigated. *Lotus burtii* does not require any of the *nodD* genes except for *nodD1*. In contrast, *L. japonicus* receives contributions from all the *nod* genes except for *nodD4* for nodulation, because with the other *nodD* mutants a reduced SDW was observed (Table 1).

The most intricate pattern of responses in nodulation described so far is that for *Sinorhizobium meliloti*, which utilizes the three copies of *nodD* to optimize nodulation of each of its legume hosts [27]. However, now we present a regulatory pattern that involves from one *nodD* gene (leucaena, *L. burtii*) to four (*L. japonicus*). There were also indications that *nodD* genes influence nodule effectiveness, as shown for *nodD2*, *nodD4* and *nodD5* for leucaena, and *nodD2* for *L. japonicus*. Another particularity for *R. tropici* CIAT 899 was that, in general, *nodD2* was not a repressor of any of the legumes evaluated, contrary to what happens with the broadly nodulating strain NGR 234 [30].

### Nod-factor patterns

The interesting roles of Nod factors—which apparently can go further than nodulation—have been broadly investigated over a long period of time [5, 8, 10, 12, 31]. *R. tropici* CIAT 899 synthesises a large variety of Nod factors when induced by flavonoids [19–23], or under abiotic stress conditions in the absence of flavonoids [2023]; surprisingly, Nod factors are also synthesized in the absence of any known inducer [23].

A variety of Nod-factor structures was synthesized by the *nodD3*, *nodD4* and *nodD5* mutants (Additional file 1: Table S1, Additional file 2: Table S2 and Additional file 3: Table S3). *nodD3* mutant reduced the number of Nod factors under all three conditions, control, when induced with apigenin and uneder saline conditions, while *nodD5* mutant had a decrease in the number of Nod factors under control and saline conditions (Table 2). It is noteworthy that *nodD4* increased the number of Nod factors in under salinity, without the induction of apigenin. The larger number of Nod factors was observed under saline conditions for the WT and *nodD4* mutants, whereas similar numbers were observed with apigenin (Table 2). These results add more evidence to the hypothesis that the large production of Nod factors is related to the well known properties of broad host infectivity and the high tolerance of abiotic stresses of *R. tropici*, being able to establish symbioses even under harsh environmental conditions [13–16].

**Table 2:** Number of Nod factors produced by the wild type *R. tropici* strain CIAT 899 and the *nodD3*, *nodD4* and *nodD5* mutants when grown in control B^−^ medium [20], with 3.4 μM of apigenin or salt (NaCl 300 mM). The structures of Nod factors under each condition are shown in Additional file 1: Table S1, Additional file 2: Table S2 and Additional file 3: Table S3

	B^−^ medium	Apigenin	Salt
CIAT 899 - WT	11	29	36
*nodD3*	2	21	25
*nodD4*	15	22	38
*nodD5*	8	30	26

In relation to the Nod-factor structures, the mutations in *nodD3* in general did not result in changes in the molecules produced both in the presence of apigenin and salt. However, in the negative control B^−^, the number of molecules was drastically reduced, which indicates that *nodD3* might be an activator of other regulatory genes such as *nodD1* (Additional file 1: Table S1, Additional file 2: Table S2 and Additional file 3: Table S3). Moreover, CIAT 899 produced only two Nod factors deacetyled in the presence of apigenin [V (C_18:1_) dNAc and V (C_18:1_, NMe) dNAc]. However, the *nodD3* mutant produced five deacetyled Nod factors that were not detected in the LCOs biosynthesized by the *nodD4* and *nodD5* mutants. These results suggest that the *nodD3* gene is important for the deacetylation of Nod factors produced by CIAT 899 in the presence of apigenin. Non-deacetylated Nod factors were detected in control and saline conditions (Additional file 1: Table S1, Additional file 2: Table S2 and Additional file 3: Table S3). Interestingly, it has been suggested that *hsnT* (=*noeT*) has a role in Nod-factor decoration in *Neorhizobium galegae* [32**]**.

Under saline stress, in addition to an increase in the LCOs synthesized by the *nodD4* mutant, fatty acids of C_14:1_ were not found, nor were sulphated molecules of four units of N-acetyl-glucosamine. Not least important, the strong increase in the number of molecules in the *nodD4* mutant might indicate that the gene is a repressor of other regulatory genes, such as *nodD1* (Additional file 1: Table S1, Additional file 2: Table S2 and Additional file 3: Table S3).

For the *nodD5* mutant, no quantitative or qualitative differences in LCOs were observed, whereas important differences were detected under saline stress. The mutation not only resulted in a decrease in the number of LCOs, but also the molecules included neither fatty acids C_14:0_, nor sulphated molecules of 4 units of N-acetylglucosamine. In addition, we did not detect the fatty acids C_20:0_ or C_20:1_ - V(C_20:0_, NMe, S); V(C_20:1_, NMe) and V(C_20:1_, NMe,S), found in the WT, *nodD3* and *nodD4* strains in the presence of salt. It is worth mentioning that *nodD1* and *nodD2* mutants also do not produce these factors in the presence of salt [23]. Fatty acids C_20:0_ or C_20:1_ under salt stress and/or C_14:0_ o C_14:1_ with apigenin might have the participation of genes detected in the genome of CIAT 899 [25], such as *nodE* and *nodF* genes [33]. Consequently, the absence of these fatty acids in *nodD5* mutants under saline stress indicates that other regulatory *nodD* genes were not activated under salt, and *nodD1* and *nodD2* are strong candidates, as their mutants inhibited the synthesis of these fatty acids. We suggest that *nodD5* could be an activator of *nodD1* or *nodD2* in the presence of salt to allow the expression of genes *nodE*-*nodF*.

This intricate regulatory mechanism for the production of LCOs involving five *nodD* genes—demonstrated in our study—seems to have no parallel in other rhizobia, especially under saline stress. As the tropical conditions where *R. tropici* is abundantly found are often extreme [34], this might indicate a high degree of evolution to allow the symbioses to form and function under harmful conditions. It is also worth mentioning that several other roles have been attributed to LCOs besides being involved in early steps of nodulation. For example, there are reports that LCO effects resemble those of cytokinins [29], that they have a role in mechanisms related to defense against disease [35, 36] and seed germination [37]; therefore, the role of LCO structure in phenotype determination deserves further investigation.

### Gene expression

**Fig. 2:**
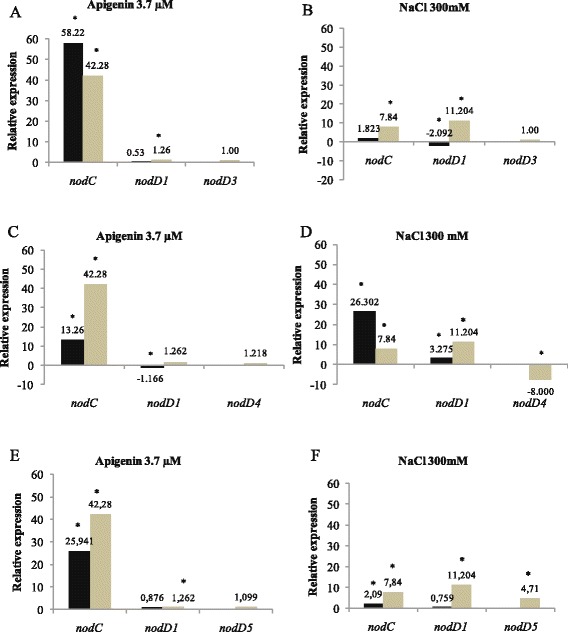
RT-qPCR analysis of the expression of *nodD* genes from *R. tropici* CIAT 899 and derivatives grown under control (B- medium), in the absence and in the presence of apigenin (3.7 μM) or NaCl (300 mM). Expression data shown are the mean (± standard deviation of the mean) of three biological replicates. Data were normalized in relation to the endogenous control (16S rRNA). The asterisks indicate a statistically significant expression at the level α = 5 %, determined by REST2009 software. Light gray bars: wild type strain, black bars: mutant. **a**, **b** - expression induced in the *nodD3* mutant; **c**, **d** - expression induced in the *nodD4* mutant; **e**, **f** - expression induced in the *nodD5* mutant

We performed gene-expression studies with the wild type and *nodD3*, *nodD4* and *nodD5* mutants. In these studies we evaluated the expression of *nodC*—which controls the elongation of the oligosaccharide chain of Nod factors and is transcribed with the activation of *nod* genes [8]—of the *nodD1* gene and of each of the three *nodD* genes, to improve our understanding of the roles of these three genes. As expected, endogenous expression of all *nodD* genes was consistently low, as these genes are constitutively expressed. In addition, the expression of each *nodD* gene corresponding to each *nodD* mutation was confirmed as null (Fig. 2).

Significant expression of *nodC* for both the WT and the *nodD3* mutant was verified, of 42- and 58-fold, respectively, when induced by apigenin, and, although at basal low levels, *nodD1* expression was significantly increased in the WT strain (Fig. 2a). Under saline stress, a mutation in *nodD3* decreased both *nodC* and *nodD1* expression, confirming the results obtained with Nod factors, that *nodD3* is an activator of *nodD1*, especially under saline conditions (Fig. 2b).

The picture obtained with *nodD4* was somewhat different. A mutation in *nodD4* resulted in a 3-fold decrease, but not in total inhibition of expression of *nodC* with apigenin; therefore, the results indicate an activation role in the presence of apigenin (Fig. 2c). However, in saline conditions, *nodD4* expression in the WT strain was down-regulated, indicating a repressor role, that was confirmed by an increase of 3.35-fold on *nodC* expression when the gene was mutated Fig. 2d. Therefore, under apigenin *nodD4* acted as an activator and under salt stress as a repressor of other *nod* genes (Fig. 2c, d).

In relation to *nodD5*, the gene proved to be an inducer, increasing the expression of both *nodC* and *nodD1* in the presence of both the inducer molecule apigenin (Fig. 2e) and salt stress (Fig. 2f). These results are consistent with the results based on the synthesis of Nod factors.

### Phylogeny of *nodD* genes

**Fig. 3:**
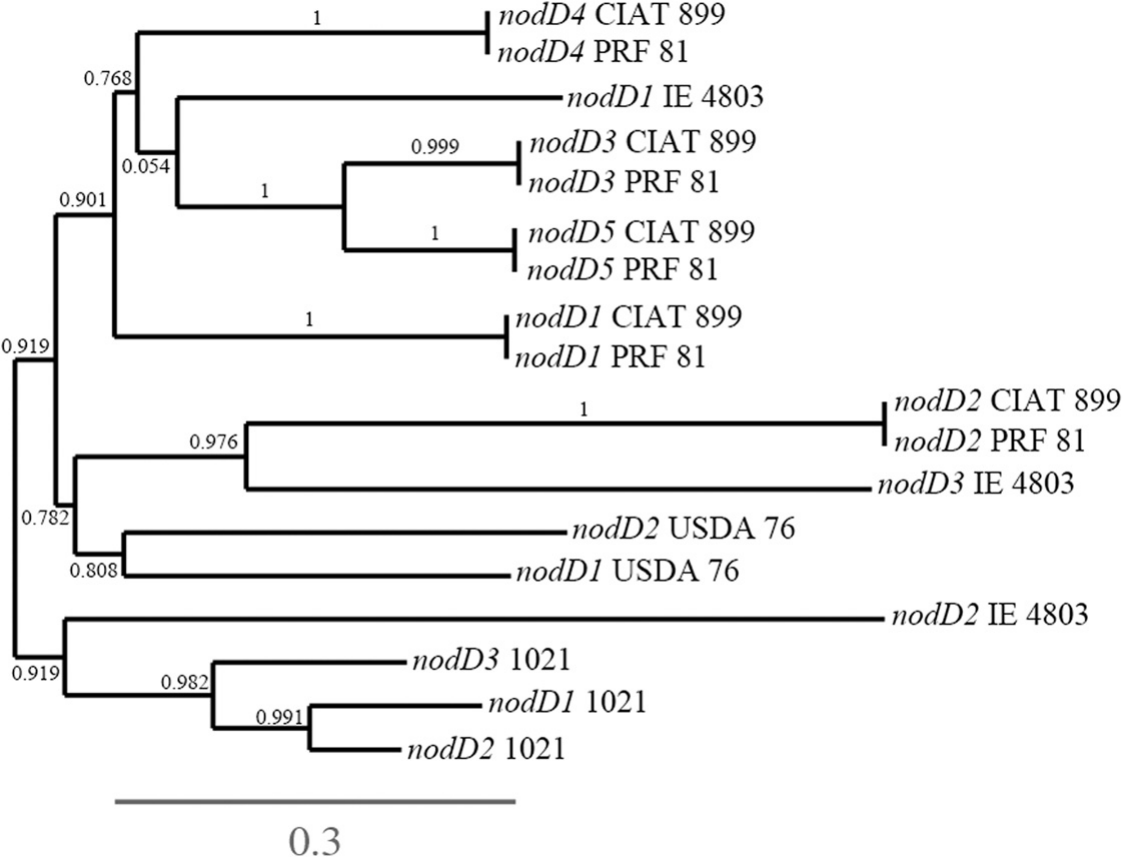
Phylogenetic tree of representatives *nodD* genes of some relevant rizobia. The branchs length represents the evolutionary lineages changing over time. The length of the brach represents the amount of changes and it is proportional to the number of nucleotide substitutions per site. The bar at the bottom of the figure provides a scale for the evolution. The numbers next to each node represent a measure of support for the node (between 0 and 1). A high value means that there is strong evidence that the sequences to the right of the node cluster together to the exclusion of any other. Phylogenetic tree was built as described in the Methods section. In this phylogenetic tree, *nodD1* to *nodD5* CIAT 899 correspond to *Rhizobium tropici* CIAT 899 *nodD* genes; *nodD1* to *nodD5* PRF 81 correspond to *Rhizobium freirei* PRF 81 *nodD* genes; *nodD1* to *nodD3* IE 4803 correspond to *Rhizobium etli* sv. phaseoli IE 4803 *nodD* genes; *nodD1* to *nodD3* 1021 correspond to *Sinorhizobium meliloti* 1021 *nodD* genes; and *nodD1* and *nodD2* USDA 76 correspond to *Bradyrhizobium elkanii* USDA 76 *nodD* genes.

A phylogenetic tree was built to verify the similarities between the five copies of *nodD* genes of CIAT 899, helping to add information about these genes. Figure 3 shows that *nodD2* gene of CIAT 899 is positioned in a different cluster from the other *nodD* genes, showing full similarity with the *nodD2* of *R. freirei* PRF 81, that belongs to the “*R. tropici* group” [38]. Within the same great cluster of the phylogenetic tree, but positioned in another subgroup was the *nodD3* genes of *R. etli* sv phaseoli. The other four copies of *nodD* genes of CIAT 899 were positioned in another great cluster, each one showing full identity with the correspondent *nodD* gene of *R. freirei*. Therefore, apparently *nodD2* had a different evolutionary history from the other *nodD* genes, and as we pointed out before, one important host legume, leucaena does not need *nodD2* for full nodulation, while common bean does. One hypothesis is that *nodD2* could have been acquired in the evolutionary process of getting the ability to nodulate common bean.

### Why five *nodD* genes?

In contrast to what we have previously reported for *nodD1* and *nodD2* [23], *nodD3*, *nodD4* and *nodD5* of *R. tropici* CIAT 899 did not affect phenotypic traits such as motility or of IAA synthesis. However, they contributed to the intricate regulatory mechanism of nodulation of different host plants that, overall, may require from one to four genes. It remains to be determined if all five genes contribute to the nodulation of common bean, since minor, but not statistically significant, decreases in nodulation were observed with *nodD3*, *nodD4* and *nodD*5 mutants.

Apparently the increased number of Nod factors and the activation of *nodD1* and *nodC* genes under abiotic stress may be critical for nodulation under abiotic stresses or for enlargement of host range, guaranteeing the survival of both symbiotic partners under harsh environmental conditions. It is surprising that one major host, common bean, releases so many *nod*-gene-inducing molecules [17, 18, 39] and that a main rhizobial symbiont synthesizes so many Nod factors by the action of five *nodD* genes. The results previously obtained with hosts such as leucaena and siratro—and now confirmed with *Lotus* spp.—where *nodD1* played the major role adds weight to the hypothesis that common bean is not the main host for *R. tropici* [23]*.* One important observation was that the phylogenetic comparisons of *nodD* genes positioned *nodD2* in a different cluster from the other *nodD* genes, what might indicate an evolution in the process of getting the ability to nodulate common bean, the requires both *nodD1* and nodD2 genes, contrary to hosts as leucaena. Sharing the nodulation responsibility with more than one *nodD* gene*,* as is the case with common bean, suggests the ultimate evolution of the symbiosis, guaranteeing that nodulation occurs under abiotic stresses.

## Conclusions

*R. tropici* CIAT 899 is an intriguing rhizobia with high tolerance of environmental stresses, the ability to nodulate a broad range of legume hosts and carrying five copies of the regulatory *nodD* gene [34]. The role of three *nodD* genes of CIAT 899 was investigated for the first time in this study. *nodD3* and *nodD5* were identified as activators of the *nodD1* gene, whereas *nodD4* might act as repressor or inducer, depending on the inducing environment. A large variety of Nod factors was produced by the three mutants when induced by apigenin or salt stress, and also in the absence of any inducer. We may hypothesize that the high number of *nodD* copies and the synthesis of many Nod factors might help *R. tropici* both in enhancing the host range and in the ability to nodulate the hosts under harsh environmental conditions.

## Methods

### Bacterial strains, plasmids, media, and growth conditions

*Rhizobium tropici* CIAT 899 and derivative strains (*nodD3*, *nodD4* and *nodD5* mutants) were grown at 28 °C on tryptone yeast (TY) medium [40], B^−^ minimal medium [20] or yeast-extract mannitol (YM) medium [41], supplemented when necessary with apigenin to a final concentration of 3.7 μM or with NaCl at 300 mM. *Escherichia coli* strains were cultured on Luria-Bertani (LB) medium [42] at 37 °C. When required, the media were supplemented with the appropriate antibiotics as described by Lamrabet et al. [43]. The same strategy described before for obtaining *nodD2* mutants of *R. tropici* CIAT 899 [23] was now used to obtain *nodD3*, *nodD4* and *nodD5* mutants by the insertion in the ORF of these genes of the Ω interposon (carrying the spectinomycin resistance gene (spc^R^ 100 μg mL^−1^). Briefly, pair primers nodD3-F (5′ – GAG CTA CCT CGA CTG CTA) and nodD3-R (5′ – CTA CCG CCA TGA TCA CCA) were used for amplifying *nodD3* gene. The 1500-bp PCR product was cloned in pGEM^®^-T Easy (PROMEGA) (Amp^R^ 100 μg mL^−1^). The PCR-amplified *nodD3* fragment was cutted with the endonuclease *Sma*I, which cut the *nodD3* gene in one site, disrupting it. The obtained DNA was ligated with Ω, which was previously digested with the *Sma*I enzyme. The ligation mixture was transformed into *E. coli* strain DH5α. The *nodD3::*Ω fragment (3,5 Kb) was excised from pGEM^®^-T Easy with the endonuclease *EcoR*I and cloned in the vector pK18mob [44], that confers resistance to kanamycin (km^R^ 30 μg mL^−1^), equally restricted with *EcoR*I.

Pair primers nodD4-F (5′ – CTG TCG CTC TGA TAT TCG A) and nodD4-R (5′ – ATA GGA CAG CCT TGG CAA) were used for amplifying *nodD4* gene. The 1497-bp PCR product was cloned in pGEM^®^-T Easy. The PCR-amplified *nodD4* fragment was excised from pGEM^®^-T Easy with the endonuclease *EcoR*I and cloned in the vector pK18mob equally restricted with *EcoR*I. In order to eliminate a *Sal*I site in the polylinker of pK18mob, the plasmid was cut with *Sma*I and *Hin*dIII and religated. The plasmid containing the PCR-amplified *nodD4* fragment was cut with the endonuclease *Sal*I, which disrupt the *nodD4* gene in one site and then was treated with the Klenow enzyme. The obtained DNA was ligated with the Ω interposon, which was previously digested with the *Sma*I enzyme. The ligation mixture was transformed into *E. coli* strain DH5α.

Pair primers nodD5-F (5′ – GCT CTT TCT TTC CCA CCA A) and nodD5-R (5′ – GAT CTG CCG ATG GCT CA) were used for amplifying *nodD5* gene. The 1478-bp PCR product was cloned in pGEM^®^-T Easy. The PCR-amplified *nodD5* fragment was excised from pGEM^®^-T Easy with the endonuclease *EcoR*I and cloned in the vector pK18mob equally restricted with *EcoR*I. This plasmid was digested with the enzyme *Xho*I, which cut the *nodD5* gene in two sites, releasing a fragment of approximately 18 pb. Rest of the plasmid was treated with Klenow enzyme to convert the cohesive end generated by the enzyme to a blunt end. The obtained DNA was ligated with the Ω interposon, which was previously digested with the *Sma*I enzyme (blunt end). The ligation mixture was transformed into *E. coli* strain DH5α.

In all cases, plasmids harbouring mutation in the *nodD3*, *nodD4* and *nodD5* genes, were transferred from *E. coli* to *Rhizobium* strains by conjugation as described by Simon [45] using plasmid pRK2013 [46] as helper. The plasmid generated was used for the homogenotization of the mutated version of the *nodDs* gene in *R. tropici* CIAT 899 by using the methodology previously described [47]. The homogenotization was confirmed by DNA-DNA hybridization. For this purpose, DNA was blotted to Hybond-N nylon membranes (Amersham, UK), and the DigDNA method of Roche (Switzerland) was employed according to the manufacturer’s instructions. A scheme of the mutation generated in the *nodD3*, *nodD4* and *nodD5* genes are shown in Fig. 1.

It is worth mentioning that growth rate was not affected by mutation in *nodD3, nodD4,* or *nodD5* genes; in addition, for the target mutagenesis of *nod* genes external primers were chosen that would allow to specifically amplify both genes, what was possible because the intergenic regions flanking both genes have different sequences. Therefore, the different phenotypes observed in both mutants are caused by loss of function of these genes. The parental and mutant strains are deposited in the culture collection of the Department of Biology of the Universidad de Sevilla and at the Diazotrophic and Plant Growth Promoting Bacteria Culture Collection of Embrapa Soja (WFCC Collection # 1213, WDCM Collection # 1054).

### Identification of nod factors

Purification and LC-MS/MS analyses of Nod factors produced by *R. tropici* CIAT 899 and derivative strains grown in B^−^ minimal medium (supplemented when required with NaCl 300 mM or apigenin 3.7 μM) were performed as described previously [22].

### RNA isolation, cDNA synthesis and quantitative RT-PCR

Wild-type CIAT 899 and mutants strains were pre-cultured in 10-mL aliquots of TY medium at 100 rpm and 28 °C in the dark. After 48 h, the three strains pre-inoculated were transferred to new media and subjected to the following conditions: control (without induction), 300 mM NaCl and apigenin 3.7 μM. These new cultures were performed in triplicate under the same conditions as for the pre-cultures, 100 rpm and 28 °C in the dark, except that were grown into the exponential phase (O.D. at 600 nm of 0.5 to 0.6).

Total RNA was extraction, verification of concentration and integrity and synthesis of cDNA were performed as described before Primers for the RT-qPCR targets, genes *nodD1*, *nodD3, nodD4, nodD5* and *nodC*, were designed using Primer3Plus (http://www.bioinformatics.nl/cgi-bin/primer3plus/primer3plus.cgi/), to obtain amplicons of 50–150 bp. With the same software, a primer to 16S rRNA was obtained and applied to normalize the relative expression of the targets. To avoid unspecific alignments, the primer sequences were searched against the *R. tropici* CIAT 899 genome (http://www.ncbi.nlm.nih.gov/nuccore/440224888?report=genbank). The primer sequences and sizes of the amplified fragments are available in Additional file 4: Table S4. RT-qPCR reactions were performed as described before [23]. Rest2009 software package [48] was used to evaluate the data by providing a robust statistical analysis (*p* < 0.05). The normalization of cycle threshold (Ct) of RT-qPCR amplifications was performed based on the selected endogenous gene (16S rRNA).

### Phenotypic traits

Analysis of exopolysaccharide (EPS), lipopolysaccharide (LPS), swimming and swarming phenotypes, biofilm formation and quantification of indole acetic acid (IAA) production were perfomed as described before [23].

### Nodulation assays

For the evaluation of the symbiotic phenotypes, wild-type *R. tropici* strain CIAT 899 and *nodD1, nodD2, nodD3, nodD4* and *nodD5* mutants were grown in YM medium until a concentration of 10^9^ cells mL^−1^ was achieved, to be used as inoculum. Surface-sterilized seeds [41] were used for the assays with common bean (*Phaseolus vulgaris* L.), leucaena [*Leucaena leucocephala* (Lam.) de Wit] and siratro [*Macroptilium atropurpureum* (DC) Urb.], *Lotus burtii* Borsos, and *Lotus japonicus* (Regel) K.Larsen. Pre-germinated seeds (about 2 days after germination) were placed in sterilized pouches or Leonard jars containing N-free nutrient solution [41], with 1 mL of inoculum of each strain added and verified for nodulation capacity after 25 (common bean), 42 days (leucaena and siratro) and 50 days (*Lotus japonicus* and *Lotus burtii*) with a 16-h 25 °C/18 °C photoperiod and about 70 % relative humidity. Shoots were dried at 65 °C until constant weight was achieved, and then weighed. Experiments were performed three times.

### Phylogenetic tree construction

Phylogenetic tree was obtained by using online plataform (http://phylogeny.lirmm.fr/phylo_cgi/) [49]. Nucleotide sequences of each *nodD* gene were first aligned by MUSCLE [50] and conserved blocks were selected [51]. The phylogenetic tree was obtained by suing the maximum-likehood algorithm [52, 53] and the TreeDyn for visualization [54].

## Additional files

Additional file 1: Table S1. Nod Factor structure biosynthesized in control condition (B^−^ medium) by wild type CIAT 899 and derivatives. (DOC 45 kb) Additional file 2: Table S2. Nod Factor structure biosynthesized in the presence of apigenin (3.7 μM) by wild type CIAT 899 and derivatives. (DOC 72 kb) Additional file 3: Table S3. Nod Factor structure biosynthesized in the presence of 300 mM NaCl by wild type CIAT 899 and derivatives. (DOC 81 kb) Additional file 4: Table S4. Sequences of the primers used in the RT-qPCR and sizes of the PCR products obtained. (DOC 37 kb)
