# Supplementary material for: Opening the “black box” of nodD3, nodD4 and nodD5 genes of Rhizobium tropici strain CIAT 899
Source: BMC Genomics. 2015 Oct 26;16:864. doi: 10.1186/s12864-015-2033-z (PMC4624370; doi:10.1186/s12864-015-2033-z)
Supplement: Additional file 1: Table S1. — Nod Factor structure biosynthesized in control condition (B− medium) by wild type CIAT 899 and derivatives. (DOC 45 kb) [file 12864_2015_2033_MOESM1_ESM.doc]

**Table S1.** Nod Factor structure biosynthesized in control condition (B^-^ medium) by wild type CIAT 899 and derivatives.

| **[M+H]^+^ (*m*/*z*)** | **B_i_ ions** | **Structure^a^** | **CIAT899^b^** | ***nodD3*^b^** | ***nodD4*^b^** | ***nodD5^b^*** |
| --- | --- | --- | --- | --- | --- | --- |
| **810** | 386 | III (C_14:0_, NMe) | - | - | + | - |
| **824** | 400, 603 | III (C_16:0_) | - | - | + | - |
| **838** | 414, 617 | III (C_16:0_, NMe) | - | - | + | - |
| **850** | 426, 629, 832 | III (C_18:1_) | + | - | - | - |
| **852** | 428, 631 | III (C_18:0_) | - | - | + | - |
| **864** | 440, 643 | III (C_18:1_, NMe) | - | - | + | - |
| **1025** | 398, 601, 804 | IV (C_16:1_) | - | - | + | - |
| **1027** | 400, 603, 806 | IV (C_16:0_) | + | - | + | + |
| **1041** | 414, 617, 820 | IV (C_16:0_, NMe) | - | - | + | + |
| **1053** | 426, 629, 832 | IV (C_18:1_) | + | + | + | + |
| **1055** | 428, 631, 834 | IV (C_18:0_) | + | - | - | - |
| **1067** | 440, 643, 846 | IV (C_18:1_, NMe) | + | - | + | - |
| **1216** | 386, 589, 792, 995 | V (C_14:0_, NMe) | + | - | - | - |
| **1230** | 400, 603, 806, 1009 | V (C_16:0_) | + | - | + | + |
| **1244** | 414, 617, 820, 1023 | V (C_16:0_, NMe) | + | - | + | + |
| **1256** | 426, 629, 832, 1035 | V (C_18:1_) | + | + | + | + |
| **1270** | 440, 643, 846, 1049 | V (C_18:1_, NMe) | + | - | + | + |
| **1338** | 631, 834, 1037 | V (C_18:0_, S) | - | - | - | + |
| **1350** | 440, 643, 846, 1049, [M-80]^+c^= 1270 | V (C_18:1_, NMe, S) | + | - | + | - |

^a^ NF structures are represented following the convention (Spaink, 1992) that indicates the number of GlcNAc residues in the backbone (Roman numeral), the length and degree of unsaturation of the fatty acyl chain, and the other substituents, which are listed in the order in which they appear, moving clockwise from the fatty acid. NMe, *N*-methyl group at glucosamine non reducing residue; S, sulfate group at reducing glucosamine residue.

^b^ Symbol: + = detected; - = non detected.

^c^ These ions arise by loss of a neutral with mass 80 Da, corresponding to the loss of SO_3_.
