# Supplementary material for: Opening the “black box” of nodD3, nodD4 and nodD5 genes of Rhizobium tropici strain CIAT 899
Source: BMC Genomics. 2015 Oct 26;16:864. doi: 10.1186/s12864-015-2033-z (PMC4624370; doi:10.1186/s12864-015-2033-z)
Supplement: Additional file 4: Table S4. — Sequences of the primers used in the RT-qPCR and sizes of the PCR products obtained. (DOC 37 kb) [file 12864_2015_2033_MOESM4_ESM.doc]

**Table S4 Sequences of the primers used in the RT-qPCR and sizes of the PCR products obtained.**

| **Name** | **Sequence** | **Amplicon** |
| --- | --- | --- |
| *nodD1* F | 5'- AAGAGCTATCACAGCCGCTTAC -3´ | 116 pb |
| *nodD1* R | 5'- ACCGTGTTCGAGCAAATACC -3´ |
| *nodD3* F | 5'-ACAATAGTGATCCGGCAAGC -3´ | 103 pb |
| *nodD3* R | 5'- ATCCGGTGCATATGAGGTTC-3´ |
| *nodD4* F | 5'- TGGATGCGGGAGACATTA -3´ | 107 pb |
| *nodD4* R | 5'- GCGTAAACTTTCGGACGAGT -3´ |
| *nodD5* F | 5'- CGCGAAATGCTGTTTGAG -3 | 103 pb |
| *nodD5* R | 5'- ACATGTGCGCAGGACAAC -3´ |
| *nodC* F | 5'- CAAGCTGCGCCCTTATCTG -3´ | 127 pb |
| *nodC* R | 5'- CAAGCAACGTGTCACGGAAA -3´ |
| 16S rRNA F | 5'- ACACACGTGCTACAATGGTG -3´ | 129 pb |
| 16S rRNA R | 5'- GCGATTACTAGCGATTCCAA -3´ |
